# How *cy pres* promotes transdisciplinary convergence science: an academic health center for women’s cardiovascular and brain health

**DOI:** 10.1017/cts.2023.705

**Published:** 2024-01-03

**Authors:** Amparo Villablanca, Brittany N. Dugger, Saivageethi Nuthikattu, Joohi Chauhan, Samson Cheung, Chen-Nee Chuah, Siedah L. Garrison, Dragan Milenkovic, Jennifer E. Norman, Luca Cerny Oliveira, Bridgette P. Smith, Susan D. Brown

**Affiliations:** 1 Department of Internal Medicine, University of California, Davis, CA, USA; 2 Department of Pathology and Laboratory Medicine, University of California, Davis, CA, USA; 3 Department of Computer Engineering, University of California, Davis, CA, USA; 4 Department of Nutrition, University of California, Davis, CA, USA

**Keywords:** *Cy pres* funding mechanism, UC Davis Women’s Cardiovascular and Brain Health research center, transdisciplinary team, convergence research, implementation science, basic science and animal modeling, neuropathology, behavioral science

## Abstract

Cardiovascular disease (CVD) is largely preventable, and the leading cause of death for men and women. Though women have increased life expectancy compared to men, there are marked sex disparities in prevalence and risk of CVD-associated mortality and dementia. Yet, the basis for these and female-male differences is not completely understood. It is increasingly recognized that heart and brain health represent a lifetime of exposures to shared risk factors (including obesity, hyperlipidemia, diabetes, and hypertension) that compromise cerebrovascular health. We describe the process and resources for establishing a new research Center for Women’s Cardiovascular and Brain Health at the University of California, Davis as a model for: (1) use of the *cy pres* principle for funding science to improve health; (2) transdisciplinary collaboration to leapfrog progress in a convergence science approach that acknowledges and addresses social determinants of health; and (3) training the next generation of diverse researchers. This may serve as a blueprint for future Centers in academic health institutions, as the *cy pres* mechanism for funding research is a unique mechanism to leverage residual legal settlement funds to catalyze the pace of scientific discovery, maximize innovation, and promote health equity in addressing society’s most vexing health problems.

## Introduction and Background

To address complex scientific problems, scientists are increasingly faced with challenges of integrating diverse disciplines. We describe the use of a convergence approach to research at the intersection of heart and brain health, a frontier of critical importance to cardiovascular disease (CVD) and dementia as leading killers of women. We detail the process and resources for establishing a new research Center for Women’s Cardiovascular and Brain Health at the University of California, Davis as a model for: (1) use of the *cy pres* principle for funding of science to improve health; (2) transdisciplinary collaboration among experts from disparate disciplines to leapfrog progress, using a convergence science approach that acknowledges and addresses social determinants of women’s cardiovascular and brain health; and (3) training the next generation of diverse researchers.

### Funding Mechanism and Cy Pres Principle

The UC Davis Women’s Cardiovascular and Brain Health was established as a research Center at the University of California, Davis in the Fall of 2021 [1]. The Center was funded by a *cy pres* mechanism from a class action court settlement in California. The term *cy pres* is derived from the old French phrase, *cy pres comme possible*, which translates to “as close as possible,” and is used in the legal field to suggest the wishes of a donor or plaintiff be carried out as closely as possible when legal issues arise surrounding the distribution of funds. It was originally applied to wills, trusts, or other similar documents, but has more recently also been applied to residual court settlements [2].

Originating in 2003, the April Krueger v. Wyeth case revolved around claims the now-defunct Wyeth Pharmaceuticals misrepresented the efficacy and safety of several of their hormone replacement therapy drugs in preventing breast cancer, cardiac disease, and dementia/Alzheimer’s disease (AD). Upon settling of the court case, which spanned nearly 20 years, the presiding judge determined the residual class action settlement monies should be set aside for women’s health initiatives targeting these very diseases. The settlement funds were competitively awarded to six major California medical institutions specializing in research on the detection, treatment, prevention, and cure of CVD in women, AD and early-onset dementia, and breast cancer. The law firm representing the plaintiffs in the case solicited proposals from the six institutions and interviewed key research scientists prior to their final funding decision. The attorneys also helped develop the concept of an annual conference to allow funded researchers from the selected institutions to work collaboratively, network, and share scientific discovery.

The University of California (UC) Davis submitted an application exemplar of teamwork driven by a diverse array of expertise that ultimately led to funding for four groundbreaking projects on health issues impacting women, with a focus on women of color and from underserved communities. This successful application netted the UC Davis School of Medicine a total of $24 million in research funding for the four projects, representing all aspects targeted by the settlement award, and collectively termed the HEAL-HER projects (Heart, BrEast, and BrAin Heath Equity Research). Herein we describe one HEAL-HER team science project, the UC Davis Center for Women’s Cardiovascular and Brain Health. This innovative Center focuses on the interconnection of CVD and dementia, as well as the shared determinants of dementia and CVD risk, and may serve as a blueprint for future Centers in academic health institutions.

### Scientific Imperative for the UC Davis Center for Women’s Cardiovascular and Brain Health

There is increasing recognition of the critical association of gender and sex with disease outcomes. One of the best examples is the multimorbidity of CVD and dementia, disorders of the heart and brain that share predisposers and risk factors. CVD is a largely preventable disease and the leading cause of death for men and women [3]. Similarly, women represent the majority of patients with dementia worldwide [4]. Though women have increased life expectancy compared to men, there are marked gender disparities in the prevalence and risk of dementia and CVD-associated mortality [5]. Women are more likely to develop dementia and die from CVD and have substantially more relative risk from CVD risk factors than men [5]. The basis for these and female-male differences, and rising disease toll, are not completely understood, including the basis for health disparities by race/ ethnicity and by social determinants of health (SDoH) [6].

In addition, heart and brain health represent a lifetime of exposures and influences, and it is increasingly recognized that the same vascular risk factors that predispose individuals to heart disease (including obesity, hyperlipidemia, diabetes, and hypertension) also compromise cerebrovascular health [7]. However, although not exclusively so, vascular injury primarily contributes to heart disease and dementia by affecting different vascular beds: microvessels in the brain, and larger caliber coronary arteries in the heart [8]. Since lifetime exposure to vascular risk factors precedes the onset of both heart disease and cognitive decline by decades, early detection and control of these modifiable risk factors are the only current known targets for prevention. Further, more effective strategies [9] are needed to accelerate early diagnosis and management and combat multimorbidity. This will require expanding our understanding of the shared causes of disorders of the brain and heart by conducting targeted research on sex/gender differences at the basic science, clinical, and population health levels.

By working at the intersection of both CVD and dementia, our collaborative team at UC Davis in the new Center for Women’s Cardiovascular and Brain Health is uniquely poised to make significant advances in: (1) addressing the mechanisms and prevention of CVD, cognitive impairment, and brain health related to sex and gender across the lifespan in women from underrepresented groups; (2) addressing the impending epidemic of dementia, and sex disparities in prevalence and death by working at the intersection of both diseases; (3) using murine models to enhance our mechanistic understanding of fundamental molecular female-male differences in how CVD risk factors differentially affect the brain’s predisposition to dementia; and (4) better defining the neuropathology of brain infarcts. Advances in these realms may lead to novel approaches to the prevention of and therapeutics for CVD and dementia.

### Social Determinants of Health (SDoH) and Women’s Cardiovascular and Brain Health

SDoH are a core emphasis across the Center’s transdisciplinary work. SDoH encompass the economic, environmental, social, and psychosocial factors that influence health, accrue and interact over the life course, and can play a significant role in disease development as root causes of health disparities [10,11]. Social determinants of health frameworks posit that social stratification – including factors such as educational status and opportunity, income disparity, discrimination, and social marginalization – impacts vulnerability and exposure to disease risk by social group.

There are several leading frameworks of SDoH. Under Bronfenbrenner’s bioecological model [12–14], a number of systems of structural influence impact health and are the root causes of disparities. Recently, these macrosystem influences have been described as being interconnected and bidirectional with micro-level influences (i.e, the immediate environment that affects personal experiences over time), highlighting reciprocal interactional processes affecting health, and best captured by more recent neo-ecological theory [11]. According to the World Health Organization’s conceptual SDoH framework [15], SDoH, as nonmedical factors that influence health outcomes, are any situation or circumstance “in which individuals are born, grow, live, work, and age” [16]. In the Healthy People 2020 [17] and 2030 frameworks [18], SDoH can be classified into 5 main domains/themes including social and community context, and health and health care (e.g., access to health services, quality of care, health literacy, and health equity) [19,20]. A health equity-focused framework has also been advanced, which postulates that SDoH, as chronic everyday lived experience stressors to the biology of disadvantaged groups, impact health factors and promote disparities in outcomes, including for CVD [21].

Vis-a-vis the specific work of our Center, SDoH are integral to CVD and dementia as the incidence, prevalence, and outcomes of CVD and dementia are unequally distributed amongst individuals and communities, and persistent health disparities are well described, including for gender groups [22,23]. SDoH affect women in underrepresented racial/ethnic groups disproportionately resulting in higher CVD prevalence and risk in these groups [24]. Regarding dementia, in adults age 65 and older, the prevalence in African American/Black and Hispanic/Latino individuals is 18% and 14%, respectively, compared to 10% in White individuals, and 21.1% in women compared to 11.6% in men [25]. In addition, recent deep phenotyping of dementia performed by leveraging an exceptionally large clinical medical records data set identified sex-specific clinical variables. For example, female patients with dementia had a greater association with CVD risk factors, including hypertension and hyperlipidemia, compared to male patients who had a greater association with behavioral risk and other factors [26]. While evidence of disparities in dementia is evident for some groups, research on the upstream factors that influence these disparities still lacks clarity, and there is increasing recognition that SDoH significantly contribute to morbidity, mortality, and health inequality and as such, are fundamental drivers of disparities in both dementia and CVD. Furthermore, SDoH do not necessarily have a one-way causal relationship with health; they can be considered as upstream factors (the causes of the causes) [27]. There are also disparities in CVD occurrence and outcomes as a result of the complex and integrated relationships between SDoH and CVD. Examination of these relationships can be referred to as the biology of adversity and allostatic load and may result in chronic inflammation, sympathetic activation, immunity alterations, and other biological disruptions known to contribute to CVD [28]. For instance, low educational opportunity is associated with low health literacy and knowledge of heart-healthy habits [29]. Similarly, limited access to healthy foods, as well as built environments not conducive to physical activity, can impact a variety of health behaviors, including poor nutrition, weight, and eating patterns over the life course [30]. In this regard, there is evidence that SDoH are related to risk factors that include high blood pressure, inflammation, chronic stress, and hyperlipidemia [31–34].

One additional field of emerging study to understand environmental influences on biology and pertinent to the focus of studies in our Center involves the role of SDoH in genomics and in epigenetic processes (DNA methylation, microRNAs, and long non-coding RNAs) that regulate gene expression/suppression [35,36]. For example, the metabolic syndrome (a clustering of CVD risk factors characterized by insulin resistance and largely attributable to lifestyle) has been linked to differentially DNA methylated CpG sites on blood mononuclear cells, particularly in Hispanics with obesity and elevated triglycerides [37]. In addition, DNA methylation of SOCS3 is involved in insulin and leptin signaling in Caucasian populations with the metabolic syndrome [37,38]. Thus, the physiologic dysregulation accompanying socially determined lifestyles that contribute to the metabolic syndrome, a known risk factor with consequences for both CVD and dementia [39,40], can be linked directly to changes at the molecular level. Furthermore, research in human social genomics has begun to identify molecular pathways through which SDoH, like psychological factors and characteristics of one’s social environment, can regulate expression of genes in immune cells and consequently affect chronic disease progression, symptom development, treatment resistance, morbidity, and mortality [41,42]. These findings present a background for reducing social disparities in health by mitigating molecular risk changes before they develop into diseases, such as by interventions that seek to alter social contexts, behavioral, or family environments in early life.

**Figure 1.**
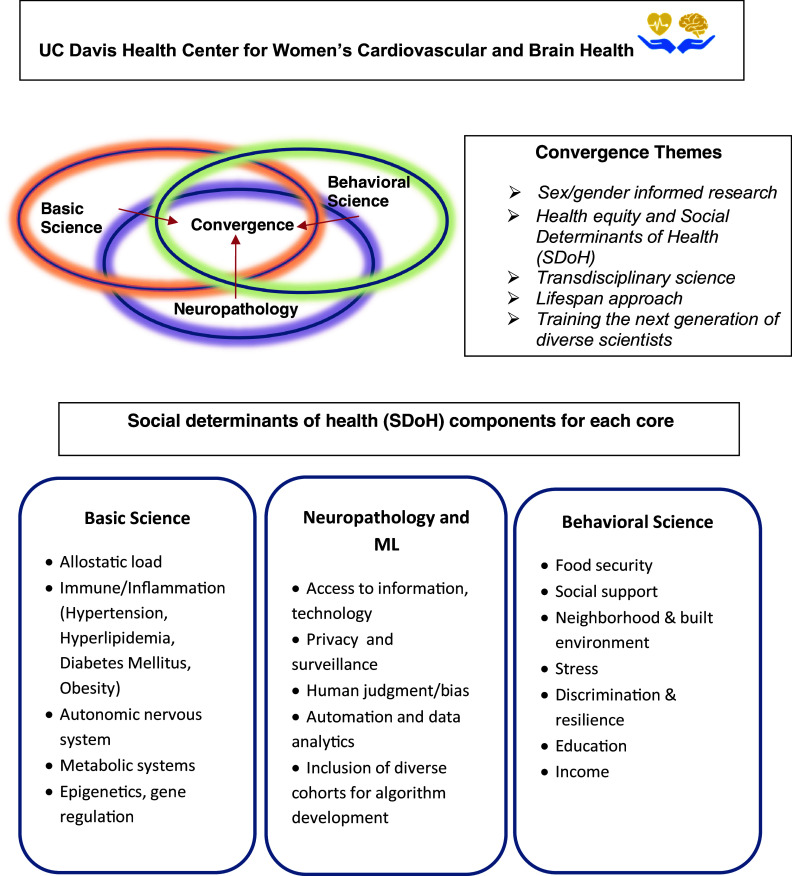
Convergence science themes and social determinants of health components of the UC Davis Center for Women’s Cardiovascular and Brain Health: basic science, neuropathology, and behavioral science cores.

Thus, an understanding of theories and frameworks of SDoH provides a broader context for our Center’s work, as described below for each core of the Center and illustrated in Figure 1, and helps its researchers and trainees understand that they are fundamental for reducing health inequities and improving health.

## Novelty of the Center

### Scientific Thematic Organization

By applying lifespan and gender perspectives to the detection, treatment, prevention, and potential cure of women’s cardiac and brain disease, the Center emphasizes the effects of these diseases on marginalized and diverse communities in California. These are areas where UC Davis has unique strength in its outreach and catchment area, as the city of Sacramento is one of the most diverse in America [43]. As such, our work has strong potential to serve those historically underrepresented in research, and also disproportionately affected by CVD and associated dementias.

### Convergence Research Science

The Center prioritizes a convergence research approach – a means for solving complex research problems, with a focus on societal needs. It entails integrating knowledge, methods, and expertise from different disciplines and forming novel frameworks to catalyze scientific discovery and innovation. With typical funding mechanisms such as the National Institutes of Health (NIH), this historically has been difficult given resources are often distributed by funding Institutes [44]. Yet, growing convergence research has been identified as one of 10 Big Ideas at the National Science Foundation each representing “areas that have been identified as ripe for rapid advancement and significant societal impacts.” These ideas require basic, fundamental research, with transdisciplinary input and teams of researchers coming together to solve complex challenges. Our Center investigators aim to adopt convergence science principles by striving to integrate their expertise, knowledge, ideas, data, and findings as conceptualized in Figure 1.

### Transdisciplinary Team and Center Leadership

**Figure 2.**
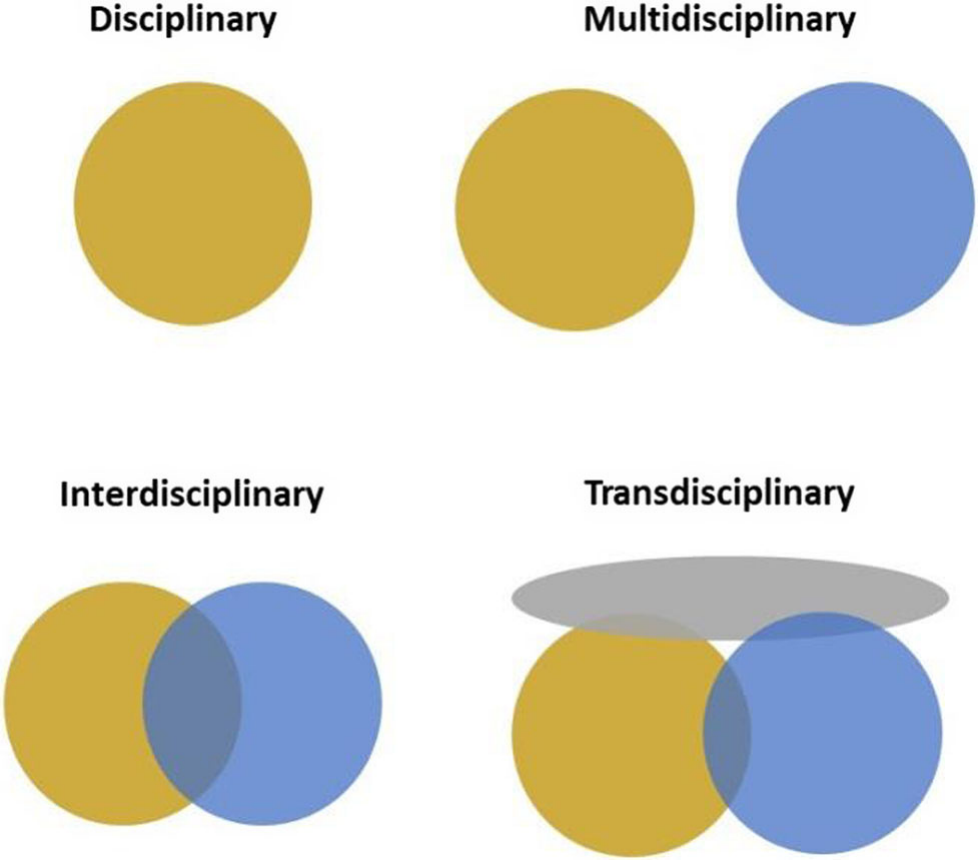
Transdisciplinary science framework: a holistic research approach that crosses disciplinary boundaries.

Conducting convergence science within the UC Davis Center for Women’s Cardiovascular and Brain Health involved assembling a transdisciplinary team from the fields of cardiovascular medicine, nutrition, computer science and engineering, neuropathology, and behavioral medicine and implementation science. In so doing, we have assembled a research team using a holistic approach that crosses disciplinary boundaries. The team is also ethno-racially diverse and diverse in career spectrum as it is composed of faculty, postdoctoral scholars, graduate students, program managers, research associates, and undergraduate students. The female predominant team shared a vision to have a deep inclusion of knowledge of the represented disciplines focused on women’s health. By taking an inclusion approach [45] to the perspective of multiple disciplines, we aim to connect new knowledge and an in-depth understanding of the focus areas of the Center to the real-life experiences of its study subjects and investigators. In addition, we seek to create a unified intellectual convergence science framework that goes beyond the disciplinary perspective, to a transdisciplinary one, as illustrated in Figure 2.

The sharing of ideas, concepts, and scientific expertise is important to collaboration across scientific disciplines. Transdisciplinary cross-fertilization needs to overcome the traditional model of partitioned research while not undermining the identity and value of individual partitions/disciplines. The Center leadership uses evidence-based practices [46] to foster ongoing cross-disciplinary fertilization and regular interaction and exchange between project cores in order to build community and guard against self-containment. These include: (1) sharing knowledge and perspectives at regular monthly team meetings, (2) stimulating creativity by associative network thinking [47] such as for behavior/biology/machine learning, (3) practicing diversity and inclusion as essential and crosscutting themes (e.g., encouraging all team members, including trainees, to provide scientific feedback on all projects across disciplines), and (4) working together (PIs/staff/trainees) on transdisciplinary scholarly work such as joint innovative manuscripts to stress overlapping concepts across cores. The Center also encourages staff (e.g, project managers) and students/trainees to work in groups for joint scientific presentations, and designing conceptual figures (such as Fig. 1). In addition, Center leadership and investigators share opportunities for professional development with the Center investigators, as well as newly published articles relevant to the Center’s work. Thus, the Center’s leadership focuses on team-based research to build synergy and leverage complementary knowledge and capabilities among scholars diverse in discipline, race/ethnicity, and career stage.

### Innovation

To our knowledge, the UC Davis Center for Women’s Cardiovascular and Brain Health is one of only a few research Centers nationally and internationally with a specific focus on the intersection of heart and brain health, and the only one with a specific focus on this intersection in women in minoritized communities. Table 1 summarizes our review of other similar research Centers generated by discussion with colleagues, review of NIH reporter, and an internet search (Google search terms: “research AND Center AND (heart OR cardiovascular) AND brain”; “research Center for heart and brain”; and “heart brain Center”).

**Table 1.** Centers dedicated to research at the intersection of cardiovascular and brain health

Center/program name	Institution	Specific Focus
**North America**		
Heart-Brain Institute	Cleveland Clinic	None mentioned
“…an institute dedicated to researching the medical interconnections between the heart and the brain.” https://philanthropynewsdigest.org/news/cleveland-clinic-receives-17-million-for-new-heart-brain-institute		
Institute for Heart and Brain Health (IHBH)	University of Michigan	None mentioned
“The mission for the institute is to become the world’s premier research institute discovering the root causes of heart and brain disease, determining how these organs communicate with each other, and developing the treatments of tomorrow” https://medicine.umich.edu/medschool/news/medical-school-establishes-institute-heart-brain-health-anthony-rosenzweig-md-inaugural-director		
Vermont Center for Cardiovascular and Brain Health (VCCBH)	University of Vermont	Sustainable research programs including early career faculty
“We are studying the vital health problems facing society: cardiovascular disease, stroke and cognitive impairment.” https://www.med.uvm.edu/heartbrainhealth/home		
Pediatric Heart and Brain Research Program	University of California, San Francisco	Development and childhood congenital heart disease
“Our philosophy is to take a longitudinal approach, beginning in fetal life, to understand and improve neurodevelopmental outcomes and quality of life in children with congenital heart disease.” https://pedheartbrain.ucsf.edu/home		
Brain, Stress, Hypertension and Aging Research Program (BSHARP)	Emory University	Aging and hypertension
“…a collaborative team of investigators and research personnel that aim to better understand the relationship between brain health and the cardiovascular system, focusing on aging and hypertension.” https://med.emory.edu/departments/medicine/divisions/geriatrics-gerontology/research/labs/bsharp/index.html		
Brain-Heart Interconnectome	University of Ottawa and affiliated research institutes, McGill University, University of Saskatchewan, and more than 45 other partners	None mentioned
“This interdisciplinary research program aims to change the fundamental disconnect between brain and heart conditions.” https://www.uottawa.ca/about-us/news-all/uottawa-research-seeks-answers-brain-heart-health-link		
*Innovation Center on Se✘ Differences in Medicine (ICON-✘)	Harvard Medical School	Sex differences in the brain and body
“The neuroimmune and vascular axes are major lines of communication across the brain and body. ICON-✘ is focusing on these pathways through the lens of hormones, genes, and metabolism to identify the causes of sex differences that cross disorders of the brain and body.” https://www.icon.mgh.harvard.edu/about		
*Ludeman Family Center for Women’s Health Research	University of Colorado Anschutz Medical Campus	Cardiovascular disease, diabetes, and mental health
“Our research of diseases that pose great risks to women - cardiovascular disease, diabetes and the intersection of mental and physical health - is making healthier, more helpful futures possible.” https://medschool.cuanschutz.edu/center-for-womens-health-research/research/research-areas		
*Barbra Streisand Women’s Heart Center	Smidt Heart Institute	Female-pattern heart disease
“playing a leading role in identifying female-pattern heart disease, developing new diagnostic tools and advancing specialized care for women.” https://www.cedars-sinai.org/programs/heart/clinical/womens-heart.html		
**Europe**		
Heart and Brain Ageing Group	The University of Oxford	Aging
“Our group investigates this heart-brain link in detail, by studying how the health of our heart and large blood vessels affect the brain and memory as we grow older.” https://www.psych.ox.ac.uk/research/heart-and-brain-research		
Heart & Brain Center Goettingen (HBCG)	University Medical Center Goettingen	None mentioned
“…research the mutual dependencies and 3 common mechanisms of cardiovascular, neurological and neuromuscular diseases in a research building specially constructed for this purpose.” https://www.umg.eu/ueber-uns/infos-medien/heart-and-brain-center-goettingen/		
**Asia**		
The Research Centre of Heart, Brain, Hormone & Healthy Aging (“HBHA”)	The University of Hong Kong Li Ka Shing Faculty of Medicine	Hormone and aging
“…aim to pursue medical problems associated with heart, brain, hormone and aging in depth, the Centre brings together the research expertise of its investigator members…” https://www.med.hku.hk/hbha/		

* Centers focused on sex differences or women’s health which include heart and brain disease.

## Thematic Approach

Traditionally, the main scientific disciplines represented by our Center have often published work and preliminary data in their own respective silos. To provide a foundation, investigators for the Center were chosen based in part on a track record of successful transdisciplinary projects as well as incorporating SDoH [48–53]. To our knowledge however, the disciplines in our Center have never before been examined in an integrated fashion from the perspective of the genesis of neurodegeneration and neurovascular inflammation and cognitive decline in male and female animal models, neuropathology and machine learning, and behavioral preventive health interventions across the lifespan. The Center for Women’s Cardiovascular and Brain Health at UC Davis aims to alter the current paradigm and involve a multi-systems line of research to advance scientific knowledge. This is unique and at the forefront of science.

Leveraging the existing expertise and capabilities at UC Davis, the Women’s Cardiovascular Medicine Program, and the research team’s robust existing expertise, we are conducting work to advance our understanding of sex differences in heart and brain health across multiple simultaneous platforms. For example, utilizing capabilities of BIG data, sophisticated bioinformatics tools, and incorporating machine learning approaches for brain tissue analysis will create innovative and integrative applications. Incorporating knowledge from new paradigms for health behavior change for modifying CVD risk factors, now understood to be common for heart disease and dementia, holds promise for altering CVD risk for younger women with gestational metabolic disorders. As such, the Center’s work has the potential for direct and long-term clinical impact on two of the major killers of women- CVD and dementia.

**Figure 3.**
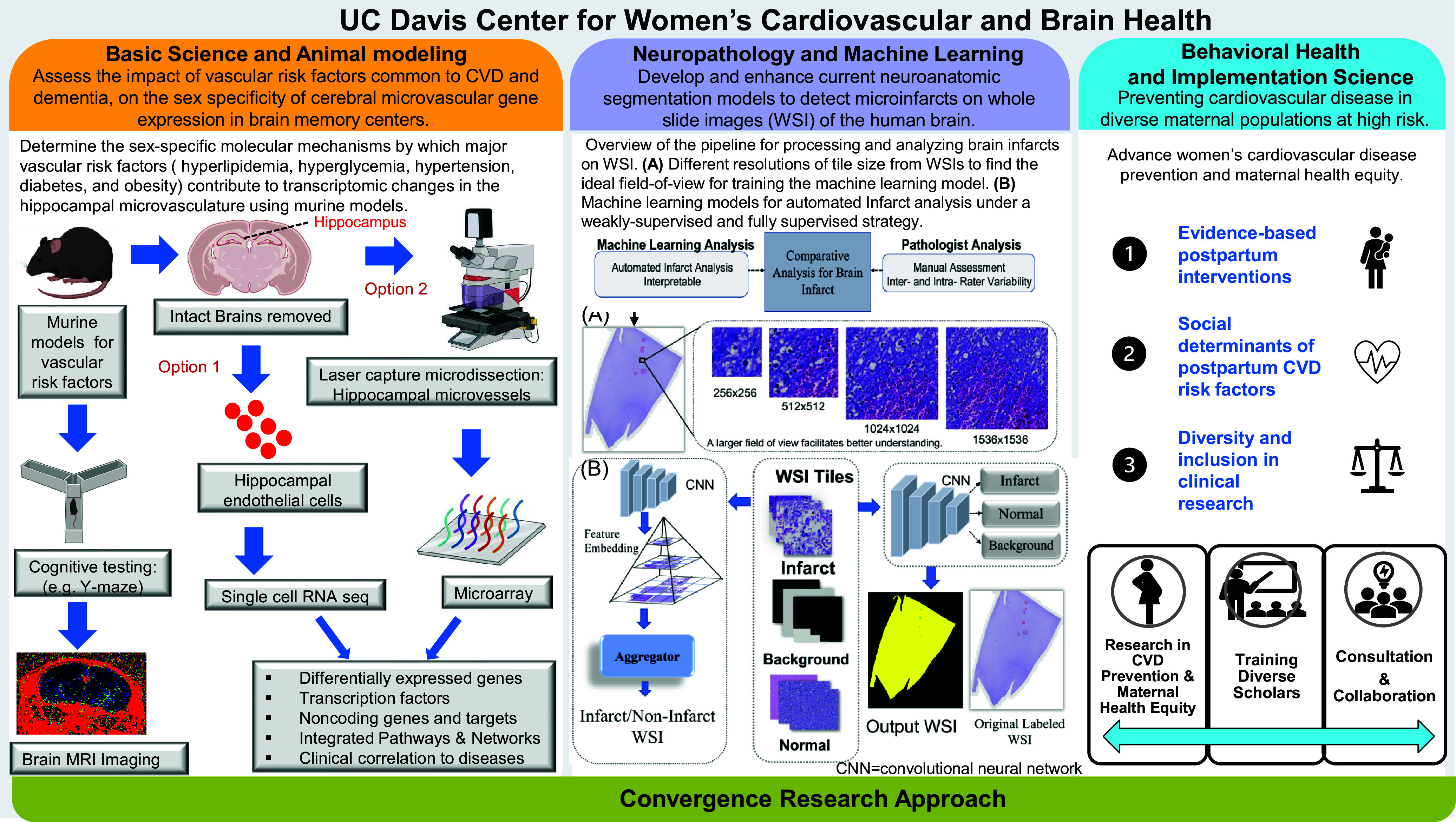
Examples of ongoing convergence science research activities of the cores of the University of California, Davis Center for Women’s Cardiovascular and Brain Health.

Our approach is of critical importance, timely, and also in close strategic alignment with national calls for research in the Center’s priority areas, including the Trans-NIH Strategic Plan for Advancing Science for the Health of Women to “leverage data sources to consider sex and gender influences that enhance research for women’s health’[54]. We have organized the Center similar to cores of an NIH-funded Center grant, and are strategically engaging three synergistic and complementary approaches as described below. Figure 3 further illustrates examples of current research activities across each core of the Center.

### Behavioral and Implementation Science Core

The Behavioral core of the UC Davis Center for Women’s Cardiovascular and Brain Health integrates behavioral science and implementation science to advance women’s CVD prevention and maternal health equity. Core activities span three pillars: research, research training, and research consultation, each with its own sub-areas of focus. Within the research pillars, the first focus is on identifying and disseminating evidence-based postpartum interventions to implement into clinical practice, with the goal of mitigating the elevated CVD risk that arises from adverse pregnancy outcomes (APOs). A major emphasis is on women with gestational diabetes (GDM), an APO that affects up to 13% of pregnancies [55]. Women diagnosed with GDM are 8.3 times more likely to develop type 2 diabetes than those with normoglycemic pregnancies, and within a decade of delivery are 2.3 times more likely to experience a cardiovascular event [56,57]. A second research focus is on examining social determinants of maternal risk factors for CVD, such as postpartum weight retention, in diverse populations. There is an urgent need for advances in these areas given the interplay between SDoH, behavioral risk factors, and CVD in women [24]. The third research focus is on promoting diverse participation in clinical research. This includes examining drivers of increased engagement in clinical research among underrepresented maternal populations that experience APOs. Indeed, the National Academies of Science, Engineering and Medicine has issued an urgent call to investigators and stakeholders to increase representation of women and underrepresented groups in clinical research [58]. Women from racial and ethnic minority groups face disproportionately higher risks of adverse health outcomes, making a health equity lens imperative to promote cardiovascular health for all women. SDoH factors relevant to research in the Behavioral Core include food security, neighborhood and built environment, socioeconomic status, social support, stress, discrimination, and resilience. Measurements of these factors will be examined in relation to maternal risk factors for CVD, such as postpartum weight retention.

Research training and consultation provide synergistic avenues to further expand the reach of the Behavioral core. These activities align with the critical importance of fostering diverse research teams to maximize innovation, effective clinical research, and health equity [59,60]. Mentoring is a vital aspect of this effort. Training activities emphasize mentoring diverse and underrepresented trainees across career stages, from undergraduate and pre-doctoral scholars to early career faculty. Hands-on experiences include secondary data analyses, literature reviews, developing data collection and stakeholder engagement tools, and presenting at the annual April Krueger Women’s Health Symposium of *cy pres* funded investigators, described below. Diverse scholars also receive advice in established research training institutes relevant to behavioral clinical trials, chronic disease prevention, and implementation science. Finally, scientific consultation activities occur within and outside of UC Davis, with a focus on interdisciplinary and transdisciplinary collaboration to advance novel approaches to research and training in women’s CVD prevention. Dissemination activities include local and national outreach, presentations, and publications, further described below.

### Neuropathology and Machine Learning Core

A relative decline in human brain tissue research has contributed to the so-called “Valley of Death” in the study of neurodegenerative diseases like dementia [61]. The Neuropathology and Machine Learning core leverages machine learning (ML) technologies, coupled with human brain samples from the UC Davis Alzheimer Disease Research Center (ADRC), to develop an automated objective means of brain micro infarct detection. Brain micro infarcts are amongst the most common cerebrovascular pathology of aging and are strongly associated with future risk of stroke and cognitive impairment [62,63]. Histologically, infarcts are typically assessed by a neuropathologist through postmortem examination of a decedent’s brain. The neuropathologist/expert classifies the infarct(s) by size (large, lacunar, or microinfarct), age (interval between onset of stroke and death-acute, subacute, chronic), location within the brain, and the presence or absence of a significant hemorrhagic component [64]. Despite published guidelines, there can be much inter- and intra-rater variability with these assessments [65,66]. Hence, there is need for a method to augment the ability of neuropathologists/experts to provide more objective quantitative evaluations and facilitate deep phenotyping.

Machine learning (ML) methods have been utilized to aid in other realms of neuropathology. This includes detection of amyloid deposits in AD and white matter/gray matter segmentation [48,67,68]. The Neuropathology and Machine Learning core seeks to adapt these pipelines and develop workflows to detect microinfarcts in postmortem human brain tissue. Additionally, we aim to benchmark performance of other ML models and frameworks to the microinfarct detection problem [69,70]. With ML, it is imperative to have generalizable data to train, test, and validate algorithms. This can help in developing robust artificial intelligence (AI) algorithms that can achieve optimal performance when applied to real-world data. Hence, we are curating a dataset utilizing materials from the ADRC brain bank. This dataset includes decedents from an array of ethnic and racial backgrounds, as well as having both women and men [71]. We also aim to explore various visualization tools to improve the interpretability of our ML models.

Investigators within the neuropathology and ML core have published on ethno-racial differences across dementia as well as highlighted the paucity of minority representation in neuropathological research [50,72,73]. Hence, the team strives to include biospecimens from underrepresented groups and analyze data accordingly if the sample size permits. The team is cognizant that the field of biomedical ML includes our access to information and technology, maintenance of donor privacy and surveillance, and seeks to minimize human judgment/bias in the design of algorithms.

It is known that cerebral small vessel disease contributes to stroke, cognitive impairment, and dementia and is highly prevalent, particularly in marginalized communities [74]. Interestingly, SDoH have also been shown to lead to distinct epigenetic signatures that potentially mediate the biological effect of environment on cardiovascular risk factors [74]. Although in the neuropathology core, the focus is mainly on developing an ML method to improve detection of cerebral infarcts in human postmortem biospecimens from the ADRC, team investigators will attempt to relate the findings to clinical variables and SDoH variables in the available databases. Lastly, the development and application of novel approaches of the Center regarding novel AI/ML may provide an opportunity to incorporate causal principled models [75] to further explore cause/effect relationships of SDoH and health disparities in our work.

### Basic Science, Animal Modeling, and Multiomics Core

The promise of increasingly powerful tools to accelerate the pace of discovery through the use of genomics, transcriptomics, gene expression profiling, and metabolomics is an exciting dimension in the Center’s research. The Basic Science, Animal Modeling, and Multiomics core is deploying well characterized, existing, and relevant experimental animal models of vascular risk factors common to CVD and dementia including: hyperlipidemia (utilizing high-fat diets and low-density lipoprotein, LDL-R -/- as well as ApoE-/- knockout models) [76,77]; hyperglycemia (utilizing high glycemic diets) [78]; hypertension (utilizing BPH/2J mice) [79]; type 2 diabetes mellitus (*db/db* mice) [80]; and obesity (*ob/ob* mice) [81]. Our focus is on the hippocampus as a key brain memory center [82], and the hippocampal microvasculature, as microvascular dysfunction is associated with cognitive impairment such as that seen in vascular dementia (VaD) [83]. As noted by the AD network, there is growing evidence that biological sex plays a significant role in dementia risk, as well as its development and progression [84]. However, the molecular mechanisms of how these CVD risk factors and biological sex contribute to microvascular injury and cognitive decline are not known or poorly understood.

The Basic Science and Animal Modeling core aims to apply animal modeling to multiomics by analyzing the brain hippocampal microvasculature from murine models of vascular risk factors using laser capture microscopy and/or single-cell RNA technology, coupled to sophisticated high throughput molecular multiomics, to study changes in differential gene expression, gene networks, pathways, transcription factors and their targets, in both female and male animal models; characterize functional outcomes by assessing cognitive performance and blood brain barrier integrity by brain gadolinium enhanced MRI; assess sex differences in the whole brain metabolome; and correlate the murine molecular gene expression profiles with published profiles of persons with AD and VaD.

To date, the work of the Basic Science and Animal Modeling core has demonstrated that both hyperlipidemic and hyperglycemic stress induces sex-specific expression of protein coding and non-coding genes [85–90]. Interestingly, the functional cellular pathways are primarily associated with neurodegeneration in male [89], and neuroprotection in female mice [88]. Furthermore, and perhaps as a result, hyperlipidemia leads to less severe cognitive dysfunction in females when compared to males [90]. Hence, our work may have significant implications for sex-specific molecular therapeutic targets for vascular risk factor-induced microvascular dysfunction associated with dementia, CVD, and cardiometabolic diseases in females and males.

SDoH have been reported in the context of cerebral small vessel disease and epigenetics [74]. Interestingly, mouse models of disease have also been employed to characterize the effect of SoDH experimentally. For example, allostatic load (the effect of chronic stress on health) has been shown to lead to shortened lifespan, elevated levels of cellular senescence markers, and increased CVD risk [91,92]. We are exerting and studying the basic science impact of a variety of metabolic stresses experimentally with use of our animal models for hypertension, hyperlipidemia, diabetes, and obesity. Additional domains of SDoH anticipated to intersect with the Animal Modeling core include activation of immune/inflammatory pathways in response to the CVD risk factors being studied in our metabolic modeling systems (hypertension, hyperlipidemia, diabetes, obesity), as well as the impacts we will be directly investigating on epigenetics and gene regulation with our multiomic studies.

## Center Scholarship, Visibility, External Engagement, and Evaluation Plans

Figure 3 represents a poster of our convergence science approach, and provides examples of ongoing research activities of each of the cores of the Center. An example of research exemplifying the convergence science principle is work we have already published [85] or have in preparation that converges our murine multiomics and cognition data with clinical data sets for patients with AD and vascular dementia (Table 2) to identify genes in common that may serve as therapeutic targets. We are also exploring a joint manuscript to converge behavioral risk factor and prevention evidence from the Behavioral and Basic Science cores.

**Table 2.** The UC Davis Center for Women’s Cardiovascular and Brain Health dissemination activities: selected local and national outreach, presentations, and scholarly work

Press Releases, UC Davis (internal communications)
2022 Press release, UC Davis Insider 2022 Newsletter, UC Davis Health Center for Translational Sciences (CTSC) 2022 Newsletter, Dept of Internal Medicine
**Publications (peer reviewed)**
Manuscripts (selected)
2023. From Basic Science to Prevention: Modifying Health Behaviors for Cardio- and Cerebrovascular Risk Reduction and Health Equity (in preparation) 2023. Reassessing Cerebral Infarcts: The Intersection of AI and User Experience in Multimodal Imaging (in preparation) 2023 Norman JE, Nuthikattu S, Milenkovic D, Villablanca AC. Sex Modifies the Impact of Type 2 Diabetes Mellitus on the Murine Whole Brain Metabolome. Metabolites. 2023 Sep 14;13(9):1012. doi: 10.3390/metabo13091012. 2023. Sex-Specific Response of the Brain Free Oxylipin Profile to Soluble Epoxide Hydrolase Inhibition. https://doi.org/10.3390/nu15051214 2022.High Glycemia and Soluble Epoxide Hydrolase in Females: Differential Multiomics in Murine Brain Microvasculature. https://doi.org/10.3390/ijms232113044 2022. The brain’s microvascular response to high glycemia and to the inhibition of soluble epoxide hydrolase is sexually dimorphic. https://doi.org/10.3390/nu14173451 2022. A sucrose diet modifies brain oxylipins in a sex-dependent manner. https://doi.org/10.1016/j.plefa.2022.102506 2022. Improving maternal cardiovascular health in underserved populations: A narrative review of behavioral intervention trials targeting postpartum weight retention. https://doi.org/10.1007/s11883-022-01045-3 2021. Inhibition of Soluble Epoxide Hydrolase Is Protective against the Multiomic Effects of a High Glycemic Diet on Brain Microvascular Inflammation and Cognitive Dysfunction. https://doi.org/10.3390/nu13113913
Abstracts
2023 Women’s cardiovascular health: Risk factors, opportunities for prevention, and novel interventions across the lifespan. https://doi.org/10.1093/abm/kaad011 2022 Effects of a High Glycemic Diet and Soluble Epoxide Hydrolase Inhibitor on Hippocampal Microvascular Function, and Multigenomic Modifications. https://doi.org/10.1093%2Fcdn%2Fnzac078.014
**Presentations (selected)**
2023 The UC Davis Center for Women’s Cardiovascular and Brain Health. April Krueger Annual Women’s Health Symposium. San Diego, CA. Sex-differences in the brain oxylipin profile. Norman JE, Nuthikattu S, Milenkovic D, Rutledge J, Villablanca AC. Cardiovascular disease prevention in diverse maternal populations at high risk: Leveraging behavioral and implementation science in the UC Davis Center for Women’s Cardiovascular and Brain Health. Smith BP, Garrison SL, Brown SD. 2023 Computationally Efficient AI frameworks for Neuropathology Image Analysis. 8^th^ annual UC Davis Postdoctoral Research Symposium. University of California, Davis Chauhan J, Oliveira LC, Lai Z, Chuah CN, Dugger BN.2023 Cardiovascular and Brain Health in Women. University of California, Davis Team Research Forum. UC Davis HEAL-HER Center for Women’s Cardiovascular and Brain Health team. 2023 Glowing Voices: Elevating the voices of diverse women with gestational diabetes to explore perspectives on clinical research. Network of Minority Health Research Investigators West Region Workshop. Las Vegas, NV. Smith, B., Greenberg, M., Scandurro, A., Serrato-Bandera, H., Millman, A., Garrison, S., Ferrara, A., Brown, S.D 2022 The Brain’s Microvascular Response to High Glycemia and to the Inhibition of Soluble Epoxide Hydrolase is Sexually Dimorphic. National Conference on Women’s Health and Sex Differences Research. Colorado Springs, CO. Nuthikattu S, Milenkovic D, Norman JE, Rutledge J, Villablanca AC. 2022 Differential Multigenomic Effect of Soluble Epoxide Hydrolase Inhibition on the Hippocampal Brain Microvasculature of Female Mice on Low and High Glycemic Diets. Gordon Research Conference on Molecular and Cellular Neurobiology. Ventura, CA. Nuthikattu S, Milenkovic D, Norman JE, Rutledge J, Villablanca AC. 2022 Effects of a High Glycemic Diet and Soluble Epoxide Hydrolase Inhibitor on Hippocampal Microvascular Function, and Multigenomic Modifications. Nutrition 2022 Live, American Society for Nutrition. Milenkovic D, Nuthikattu S, Norman JE, Rutledge J, Villablanca AC.

It is important that as a unique Center we publicize Center activities and increase visibility, present and share our work at scientific venues including meetings and symposia, engage in scholarly science for publication in peer-reviewed journals, engage external groups, and evaluate our progress yearly by tracking these metrics. Table 2 provides highlights of progress to date on the Center’s visibility and scholarship.

Regarding external engagement, all six California institutions funded by this *cy pres* mechanism convene yearly for an annual symposium, the first of which was held in May 2023 at the University of California, San Diego. The purpose of the annual convening is to share ideas, create synergy, and explore opportunities for further collaboration. We have also partnered with the Dean’s office and Center for Translational Science at UC Davis Health to deliver a virtual presentation reaching a broad audience outside the School of Medicine resulting in several novel ideas such as for a joint cardiovascular/brain biorepository. Potential future directions for external engagement with stakeholders outside academia to enrich the Center’s work include: (1) exploring public-facing interactions with scientific societies, (2) a bidirectional exchange with the California Dept of Health Services and the Office of the State’s Surgeon General in raising awareness of the intersection of heart disease and dementia, (3) engaging with our extensive community network as ambassadors of the heart/brain care message for prevention, and (4) educational enhancements of existing health system websites for patients and community groups. Future directions may also include creating a research collaborative network among this and other existing Centers (Table 1) to exchange knowledge and share lessons learned to help catalyze further progress in this field.

Success of the Center is closely tracked by leadership and stakeholders, including Center PIs; the UC Davis School of Medicine Dean’s Office, which administers the award; and the US District Court (through Class Counsel), which receives annual progress reports from all institutional recipients of these cy pres funds. The Center’s progress and impact will be tracked by a number of metrics including: local, regional, and national presentations at scientific conferences; peer-reviewed publications and abstracts; sharing and use of data deposited to public databases (such as GEO, a public functional genomics data repository); and success in securing additional funding. Although not initially included in our award, we now capitalize on the opportunity to also track the Center’s success in training the next generation of diverse researcher trainees by tracking their career progression. Opportunities to assess external engagement include the number of stakeholders with whom we are engaging and the reach of our joint activities (e.g., attendees at events, development of new educational materials, etc.).

## Conclusions, Impact, and Future Directions

The UC Davis Center for Women’s Cardiovascular and Brain Health capitalizes upon transdisciplinary knowledge, and the existing robust expertise and capabilities at UC Davis, to substantially expand discovery and impact in a cutting-edge area of science. The behavioral studies have strong potential to improve health promotion and prevention of CVD risk factors, and our understanding of how they interplay throughout the lifespan in our target population of diverse women and underserved communities. The neuropathology and machine learning studies will provide more objective tools that will aid in future studies to evaluate associations of neuropathology with sex and SDoH. The animal modeling studies for cardiovascular and dementia risk factors reveal the sex-dependent molecular mechanisms by which vascular risk factors contribute to multiomic gene changes in the microvasculature of brain memory centers that may provide critical targets for clinical therapeutics. Furthermore, a crosscutting area of impact of our Center is diversifying the research workforce by developing a pipeline of highly trained scholars as content experts in the field, critical to advancing and sustaining new knowledge on sex and gender at the intersection of heart and brain health.

The work of Center scientists holds promise to advance our understanding of persistent knowledge gaps on how CVD risk factors (diet, obesity, diabetes, and hypertension) interplay as common causative risk factors at the intersection of heart and brain health. By deploying strategies in behavioral and implementation science, neuropathology and machine learning, basic science animal modeling, and high throughput bioinformatics, we will identify sex and gender-specific mechanistic pathways enabling potential therapeutic and behavioral interventions for reducing neuro-cardio-vascular risk for women and minorities. The integrated, life course, and transdisciplinary approach of the Center, in concert with a SDoH context, is innovative with unique potential for accelerating the pace of discovery in the field. As such, the convergence science transdisciplinary model established by the Center is one that can be replicated by other institutions with similar goals and funding mechanisms. Indeed, as a new frontier in team science, convergence research holds promise to enhance the approach to discovery of some of the most vexing and complex clinical problems. Lastly, the use of the *cy pres* mechanism for funding scientific research is a model to be considered in the future to provide the substantial support needed to catalyze the pace of scientific innovation.
